# Frequencies and Specificities of “Enzyme-Only” Detected Erythrocyte Alloantibodies in Patients Hospitalized in Austria: Is an Enzyme Test Required for Routine Red Blood Cell Antibody Screening?

**DOI:** 10.1155/2014/532919

**Published:** 2014-03-25

**Authors:** Dietmar Enko, Claudia Habres, Franz Wallner, Barbara Mayr, Gabriele Halwachs-Baumann

**Affiliations:** ^1^Department of Laboratory Medicine, Central Hospital Steyr, Sierningerstraße 170, 4400 Steyr, Austria; ^2^University of Applied Sciences for Health Professions Upper Austria, Bachelor Program Biomedical Science, Central Hospital Steyr, Sierningerstraße 170, 4400 Steyr, Austria

## Abstract

The aim of this study was to determine the frequencies and specificities of “enzyme-only” detected red blood cell (RBC) alloantibodies in the routine antibody screening and antibody identification in patients hospitalized in Austria. Routine blood samples of 2420 patients were investigated. The antibody screening was performed with a 3-cell panel in the low-ionic strength saline- (LISS-) indirect antiglobulin test (IAT) and with an enzyme-pretreated (papain) 3-cell panel fully automated on the ORTHO AutoVue Innova System. The antibody identification was carried out manually with an 11-cell panel in the LISS-IAT and with an enzyme-pretreated (papain) 11-cell panel. In total 4.05% (*n* = 98) of all patients (*n* = 2420) had a positive RBC antibody screening result. Of them 25.51% (25/98) showed “enzyme-only” detected specific or nonspecific RBC alloantibodies. Rhesus and Lewis system antibodies were found the only specificities of “enzyme-only” RBC alloantibodies: all in all 4.8% (4/98) were detected with anti-E, 3.06% (3/98) with anti-Le^a^, 3.06% (3/98) with anti-D after anti-D prophylaxis and 1.02% (1/98) with anti-e. In total, 14.29% (14/98) showed a nonspecific RBC alloantibody result with the enzyme test. The results of the present study demonstrate that a high number of unwanted positive reactions with the enzyme technique overshadows the detection of “enzyme-only” RBC alloantibodies. (Trial Registration: K-37-13).

## 1. Introduction

Pretransfusion blood grouping, red blood cell (RBC) antibody screening, and compatibility testing are essential to prevent incompatible blood transfusion and alloimmunization. The Nobelist Karl Landsteiner, discoverer of the first human marker locus, published the results of a complete cross testing of the RBCs and sera of six people (including himself) in his 1901 paper [1–3]. Since then numerous other human blood group antigens have been described and categorized. Alloimmunization can cause a hemolytic transfusion reaction in individuals lacking the corresponding blood group antigen on their erythrocytes [4, 5]. RBC alloimmunization correlates with the number of transfusions [6–8], and the immunogenicity of the blood group antigens is crucial [5, 9]. About 25–28 antigens are known to cause hemolytic transfusion reactions and should be detected with the pretransfusion RBC antibody screening test [10]. The Rhesus (Rh), Kell (K), Duffy (Fy), and Kidd (Jk) antigens are some of these clinically significant blood group antigens [11].

Pretransfusion compatibility testing involves ABO grouping, Rh typing, RBC antibody screening, RBC antibody identification, and also cross matching the RBC unit designed to be transfused [12]. Hemagglutination is still the classical method for antigen testing and antibody screening [13]. The indirect antiglobulin test (IAT) is considered to be a reliable and effective method to detect clinically relevant RBC alloantibodies [14]. In the last few years, pretransfusion testing practices have shifted from tube to gel technology. The gel test is more sensitive than the conventional tube method [15, 16]. It has been well known for a long time that the enzyme treatment of RBCs modifies the erythrocyte surface [17–19] and that some Rh antibodies occur only in the enzyme (papain) technique [20, 21]. The main argument for the use of the enzyme technique in the routine testing would be to detect clinically significant RBC alloantibodies, but published works on this topic are rare [22]. In Austria, hospital blood banks without donation, production and screening facilities, and the so-called “blood-depositories” are mainly managed by specialists for anesthesiology, laboratory medicine, transfusion medicine, and internal medicine [23]. Among the blood-depositories, differences of opinion exist regarding the use of enzyme-pretreated RBCs.

The aim of this study was to determine the frequencies and specificities of “enzyme-only” detected erythrocyte alloantibodies in the routine RBC antibody screening and identification in patients hospitalized in Austria.

## 2. Materials and Methods

The ethical approval for this study was provided by the Ethical Committee of Upper Austria, Linz, Austria (Trial Registration no.: K-37-13). The study period was from January 17, 2013 to May 17, 2013.

### 2.1. Patient Material

Blood samples of 2420 hospitalized patients, who underwent routine blood grouping and RBC antibody screening at the Department of Laboratory Medicine in the Central Hospital Steyr (Austria), were investigated. The patients were mainly adults and in very few instances children. Ethylendiamintetraacetic acid (EDTA) plasma was used for the analysis. All the patients were tested for the ABO blood group, the Rh antigen D, and RBC alloantibodies.

### 2.2. Blood Group Determination

The fully automated ABO/Rh typing was performed using the gel technique on the ORTHO AutoVue Innova System (Ortho Clinical Diagnostics, Raritan, New Jersey). According to the Austrian Guidelines for Blood Group Serology and Transfusion Medicine (latest version July 2000), the RBC antigens A, B, and Rh D, as well as the presence of isoagglutinins, were tested. All patients with a positive result in the RBC antibody screening were tested for the Rh antigens C, c, E, and e.

### 2.3. RBC Antibody Screening

The RBC antibody screening was carried out in a fully automated process with a 3-cell panel (0.8% Surgiscreen RBCs reagents 1, 2, and 3 [Ortho Clinical Diagnostics, Raritan, New Jersey]) in the low-ionic strength saline- (LISS-) IAT. Additionally an enzyme test with 3 enzyme-pretreated (papain) red test cells (ID-DiaCell PI, PII, PIII [BIO-RAD, Hercules, California]) was performed on the ORTHO AutoVue Innova System (Ortho Clinical Diagnostics, Raritan, New Jersey).

### 2.4. RBC Antibody Identification

If the RBC antibody screening showed a positive result in the LISS-IAT and/or with the enzyme-test (papain), the antibody identification was carried out manually with an 11-cell panel (ID-DiaPanel 1-11 [BIO-RAD, Hercules, California]) in the LISS-IAT and with an enzyme-pretreated (papain) 11-cell panel (ID-DiaPanel-P 1-11 [BIO-RAD, Hercules, California]). If a specific RBC alloantibody was identified, the corresponding blood group antigen was determined to confirm the RBC antibody identification result. The autocontrol was made in the LISS-IAT. Serological reactions were graded from 0 to 4+.

### 2.5. Data Analysis

Descriptive statistics were performed to analyze the serological ABO and Rh phenotype frequencies and the frequencies and specificities of the RBC alloantibodies.

## 3. Results

### 3.1. ABO and Rh D Phenotype Frequencies

Of all patients (*n* = 2420), 38.18% (924/2420) had blood group O, 44.42% (1075/2420) blood group A, and 12.44% (310/2420) blood group B and 4.96% (120/2420) showed the AB phenotype. All in all 84.42% (2043/2420) were Rh antigen D positive and 15.58% (377/2043) were Rh antigen D negative.

### 3.2. RBC Antibody Screening Results

In total 4.05% (*n* = 98) of all patients (*n* = 2420) showed a positive RBC antibody screening result with the 3-cell panel in the LISS-IAT and/or with the enzyme-pretreated (papain) 3-cell panel. Of them 82.65% were women and 17.35% were men. The median age of patients with a positive RBC antibody result was 42 (range: 17–91) years. In total 95.95% (*n* = 2322) had a negative test result in the LISS-IAT as well as with the enzyme test.

### 3.3. RBC Antibody Identification Results

The frequencies and specificities of RBC alloantibodies detected in the LISS-IAT ± enzyme (papain) are illustrated in Table 1: in total, 66.32% (*n* = 65) of all patients with a positive RBC antibody screening result (*n* = 98) presented a specific RBC alloantibody identification result. The most common specific RBC alloantibodies were directed against Rhesus (Rh), Lewis (Le), and Kell (K) antigens. Six patients were found with combined specific RBC alloantibodies: three patients had combined anti-D and anti-C, one patient combined anti-D, anti-C, and anti-K, one patient combined anti-E, anti-K, and anti-Fy^a^, and one patient had combined anti-c, anti-K, and anti-Fy^a^. Eight patients were found with a nonspecific RBC alloantibody identification in the LISS-IAT ± enzyme (papain). The frequencies and specificities of “enzyme-only” detected RBC alloantibodies are shown in Table 2: all in all eleven patients showed a specific RBC alloantibody result with the enzyme test. The identified “enzyme-only” RBC alloantibodies were Rhesus and Lewis system antibodies. Fourteen patients showed a nonspecific RBC alloantibody result with the enzyme test. All specific and nonspecific RBC alloantibody detection results in the LISS-IAT and with the enzyme test are summarized in Table 3.

## 4. Discussion

The ABO and Rh D frequency data of our study population are comparable with the described results in the literature. A study about blood group frequencies in Germany with the data of more than 600,000 donors demonstrated similar results: all in all 41.21% had blood group O, 43.26% blood group A, 10.71% blood group B, and 4.82% blood group AB. 82.71% were Rh D positive and 17.29% were Rh D negative [24].

The RBC antibody screening and the cross-match are used as pretransfusion tests in the daily routine of blood banks and hospitals. Both routine tests are designed to detect agglutinating and hemolyzing erythrocyte antibodies active at 37°C [25]. In this study 4.05% (*n* = 98) of all hospitalized patients (*n* = 2420) showed a positive RBC antibody testing result. Compared to previous studies, blood type alloantibodies have been reported in 0.8% of blood donors, in 1-2% of hospitalized patients, and in 2–9% of patients with a history of blood transfusion [26].

Over the years a lot of distinct blood group antigens have been identified. Carbohydrate antigens (ABO and P/Globo-related system) and protein antigens (Kidd, Duffy, and Rh blood group system) have been described [27]. They can act as functional molecules but also can evoke erythrocyte antibodies [28]. Therefore pretransfusion RBC antibody detection and identification are essential. According to the Austrian Guidelines for Blood Group Serology and Transfusion Medicine (latest version July 2000), the RBC antibody screening must be performed as an IAT with at least two erythrocyte test cells. The completion with an enzyme test is not explicitly postulated. Despite the widespread use of the RBC antibody screening test, differences of opinion exist regarding the use of enzyme-pretreated red test cells [29, 30]. The collected data of the present study illustrate that Rhesus and Lewis system antibodies were identified as the only specificities of “enzyme-only” RBC alloantibodies. These results confirm previous studies, where anti-E and anti-Le^a^ were found to be the most frequent erythrocyte antibodies active only in the enzyme test [29, 31]. In a retrospective study on blood samples from 10,000 recently transfused patients, the majority of “enzyme-only” antibodies was not clinically significant. Of 19 patients with an “enzyme-only” erythrocyte antibody, who were transfused with antigen-positive RBC units, only one had a delayed hemolytic transfusion reaction [32]. In another work six males with naturally occurring Rh antibodies were investigated. Two subjects with “enzyme-only” Rh antibodies (one anti-E and one anti-D) in papain technique showed a normal survival of incompatible injected RBCs [33]. As far back as 1961 it was considered that Rh antibodies only detectable by an enzyme (papain) method do not play such a clinically significant role in the etiology of hemolytic reactions compared to antibodies detectable by the IAT [20].

In the present study 3.06% (*n* = 3) of all patients with a positive RBC antibody screening result (*n* = 98) showed an “enzyme-only” anti-Le^a^ in the antibody identification. In general Le antibodies are not considered clinically significant [11]. This is probably because the most of the antibodies in the Lewis system are not active at 37°C [34].

In this study a high number of unwanted positive enzyme reactions were detected. In total 14.29% (*n* = 14) of patients with a positive antibody testing result (*n* = 98) had a nonspecific RBC alloantibody identification result with the enzyme test. These findings confirm the results of other works on this topic. The detection of clinically significant “enzyme-only” erythrocyte antibodies was overshadowed by the high number of nonspecific or clinically insignificant antibodies [29]. Many specialists working on this as a daily routine are convinced that the enzyme test in the RBC antibody screening is not justified because of the high workload in investigating all the initial positive enzyme-method reactions compared with the low number of clinically significant erythrocyte antibodies identified [32].

The limitation of this study is the low number of patients. In a greater study population more “enzyme-only” RBC alloantibodies may be detected and the results of the present study could be substantiated.

## 5. Conclusions

The data presented in this study demonstrate that Rhesus and Lewis system antibodies were the only specificities of “enzyme-only” detected RBC alloantibodies in patients hospitalized in Austria accompanied by a high number of unwanted nonspecific enzyme reactions. Compared to the existing literature the majority of “enzyme-only” detected RBC antibodies are considered to play a minor part in the etiology of hemolytic transfusion reactions. In clinical practice extra time and additional personnel and labor costs for investigating a high number of unwanted positive enzyme-reactions must be calculated. Based on these facts each hospital “blood-depository” should make its own pragmatic decision whether the enzyme test for routine RBC antibody screening is justified or not in their patient population.

## Figures and Tables

**Table 1:** Frequencies and specificities of RBC alloantibodies detected in the LISS-IAT ± enzyme (papain) (*n* = 73).

Antibody-specificities	Frequencies	Percent
Anti-D after anti-D prophylaxis	32	32.66
Anti-E	3	3.06
Anti-Le^a^	4	4.08
Anti-D	5	5.10
Anti-K	5	5.10
Anti-D and anti-C	3	3.06
Anti-Fy^a^	2	2.04
Anti-Kp^a^	2	2.04
Anti-M	2	2.04
Anti-C^w^	1	1.02
Anti-S	1	1.02
Anti-D, anti-C, and anti-K	1	1.02
Anti-E, anti-K, and anti-Fy^a^	1	1.02
Anti-c, anti-K, and anti-Fy^a^	1	1.02
Anti-I	1	1.02
Anti-HI	1	1.02
Nonspecific RBC alloantibodies	8	8.17

Total	73	74.49

RBC: red blood cell; Le^a^: Lewis^a^; K: Kell; Fy^a^: Duffy^a^; LISS: low-ionic strength saline; IAT: indirect antiglobulin test.

**Table 2:** Frequencies and specificities of “enzyme-only” detected RBC alloantibodies (*n* = 25).

Antibody specificities	Frequencies	Percent
Anti-E	4	4.08
Anti-Le^a^	3	3.06
Anti-D after anti-D prophylaxis	3	3.06
Anti-e	1	1.02
Nonspecific RBC alloantibodies	**14**	**14.29**

Total	25	25.51

RBC: red blood cell; Le^a^: Lewis^a^.

**Table 3:** RBC alloantibody detection in the LISS-IAT and with the enzyme test.

*n* = 98	LISS-IAT + enzyme	LISS-IAT	“Enzyme-only”
Specific RBC alloantibodies	60	5	11
Nonspecific RBC alloantibodies	7	1	**14**

RBC: red blood cell; LISS: low-ionic strength-saline; IAT: indirect antiglobulin test.
